# Actinobacteria as Promising Candidate for Polylactic Acid Type Bioplastic Degradation

**DOI:** 10.3389/fmicb.2019.02834

**Published:** 2019-12-19

**Authors:** Natthicha Butbunchu, Wasu Pathom-Aree

**Affiliations:** ^1^Master of Science Program in Applied Microbiology (International Program), Faculty of Science, Chiang Mai University, Chiang Mai, Thailand; ^2^Graduate School, Chiang Mai University, Chiang Mai, Thailand; ^3^Department of Biology, Faculty of Science, Chiang Mai University, Chiang Mai, Thailand; ^4^Center of Excellence in Microbial Diversity and Sustainable Utilization, Faculty of Science, Chiang Mai University, Chiang Mai, Thailand

**Keywords:** actinobacteria, polylactic acid, biodegradation, PLA-degrading enzyme, serine protease, bioplastic

## Abstract

Polylactic acid (PLA) is one of the most commercially available and exploited bioplastics worldwide. It is an important renewable polymer for the replacement of petroleum-based plastic materials. They are both biodegradable and bio-based plastic. Microbial degrading activity is a desirable method for environmental safety and economic value for bioplastic waste managements. Members of the phylum actinobacteria are found to play an important role in PLA degradation. Most of the PLA degrading actinobacteria belong to the family *Pseudonocardiaceae.* Other taxa include members of the family *Micromonosporaceae, Streptomycetaceae, Streptosporangiaceae*, and *Thermomonosporaceae.* This mini-review aims to provide an overview on PLA degrading actinobacteria including their diversity and taxonomy, isolation and screening procedures and PLA degrading enzyme production from 1997 to 2019. Consideration is also given to where to sampling and how we might use these beneficial actinobacteria for PLA waste management.

## Introduction

Plastics are polymeric materials widely used globally with an important role in everyday life. Increasing demand promotes global plastic market to grow continuously (Mekonnen et al., 2013; Geyer et al., 2017). Conventional petroleum-derived plastics such as polyethylene terephthalate, PET are commonly used as single-use packaging which caused environmental problems from their resistant to degradation. Over the past few decades, awareness on our dependence on limited oil supply has been increased. These have inspired researchers all over the world to search for alternative renewable plastics. Eventually, bioplastics have been successfully developed (Vink et al., 2003, 2004). Bioplastics are large family of different materials. The word “bio-plastics” refers to either biodegradable plastics, bio-based plastics or features both properties (Tokiwa et al., 2009; Alshehrei, 2017). Bio-based is the term for material partly derived from renewable resources, while bio-degradable is a chemical process during which available microorganisms in the environments convert materials into natural substances (de Wilde, 2009; Song et al., 2009, 2011). Recently, there are bioplastic alternatives for almost all conventional plastics. Among currently available bioplastics, poly(lactic acid) (PLA) is one of the bio-based and biodegradable plastic of great commercial value (Karan et al., 2019). It is an aliphatic polyester manufactured by ring-opening polymerization of lactide or by polycondensation of lactic acid monomers derived from the fermentation of starch as a feedstock (Garlotta, 2001; Calabia et al., 2010; Karamanlioglu et al., 2017). PLA exhibited many excellent properties such as thermoplastics, gas barrier, UV resistant, elastic, rigid, hydrophobic, and biocompatible tissue (Farrington et al., 2005; Carosio et al., 2014; Scarfato et al., 2015; Farah et al., 2016). Therefore, it can be used for various applications such as food packaging and fiber suitable for technical textile application. Moreover, PLA has been used extensively in the medical field due to their ability to be incorporated into human and animal bodies as medical implants, drug delivery materials, and surgical sutures (Jalil, 1990; Ikada and Tsuji, 2000; Ahmed et al., 2018). Biodegradability of PLA is a critical consideration for the development of suitable biological treatments for waste management. A number of microorganisms and their enzymes are involved in biodegradation process especially, bacteria and filamentous fungi (Williams, 1981; Torres et al., 1996; Jarerat and Tokiwa, 2001; Kim et al., 2008; Szumigaj et al., 2008; Bubpachat et al., 2018). Actinobacteria are the first bacteria that found to degrade PLA (Pranamuda et al., 1997). This discovery opened up a view on actinobacteria as potential PLA degrading microorganisms.

## Diversity of PLA-Degrading Actinobacteria: How to Find and Where to Look?

Polylactic acid-degrading ability in microorganisms are not widely distributed as PLA degraders are less discovered than other types of plastic degrading microorganisms (Nishida and Tokiwa, 1993; Pranamuda et al., 1997; Suyama et al., 1998; Tokiwa et al., 2009). Actinobacteria are among the few microorganisms with potential for PLA degradation (Jarerat et al., 2002, 2003; Sukkhum et al., 2009a, b, 2011, 2012; Chomchoei et al., 2011; Konkit et al., 2012; Penkhrue et al., 2015, 2018; Panyachanakul et al., 2017, 2019). PLA-degrading actinobacteria are affiliated with 26 species in 11 genera namely *Actinomadura*, *Amycolatopsis*, *Kibdelosporangium*, *Micromonospora*, *Nonomuraea*, *Pseudonocardia*, *Saccharothrix*, *Streptoalloteichus*, *Streptomyces*, *Thermomonospora*, and *Thermopolyspora* (Table 1).

**TABLE 1:** List of PLA-degrading actinobacteria, their degrading enzymes and degradation detection methods from 1997 to 2019.

**Family**	**Genus**	**Species/strain**	**Sample sources**	**Isolation medium**	**Isolation method**	**Degradation detection method**	**Enzyme type**	**References**
*Micromonosporaceae*	*Micromonospora*	*Micromonospora echinospora* B12-1	Soil	Emulsified PLA agar	Plate count and clear zone	Turbidity method	–	Sukkhum et al., 2009b
		*Micromonospora viridifaciens* B7-3	Soil	Emulsified PLA agar	Plate count and clear zone	Turbidity method	–	Sukkhum et al., 2009b
*Pseudonocardiaceae*	*Amycolatopsis*	*Amycolatopsis* sp. HT-32	Soil form paddy fields, weed fields, roadsides, and dumping grounds	0.1% (w/v) emulsified PLA	Plate count and clear zone	Film-weight loss; monomer production	Protease	Pranamuda et al., 1997
		*Amycolatopsis* 3118	Not specified	Basal medium	Plate count and clear zone	Film-weight loss; monomer production	Protease	Ikura and Kudo, 1999
		*Amycolatopsis* sp. KT-s-9	Not specified	PLLA emulsified and silk plates	Plate count and clear zone	–	Protease	Tokiwa et al., 1999
		*Amycolatopsis mediterranei* ATCC 27649	Not specified	–	–	Clear zone method	–	Pranamuda and Tokiwa, 1999
		*Amycolatopsis* sp. 41	Not specified	Emulsified PLA agar	Plate count and clear zone	Film-weight loss; monomer production	Protease	Pranamuda et al., 2001
		*Amycolatopsis* sp. K104-1	Soil	0.1% emulsified PLLA	Plate count and clear zone	Turbidity method	Serine protease	Nakamura et al., 2001
		*Amycolatopsis orientalis* ssp. *orientalis*	^∗∗^IFO 12362	–	–	Film-weight loss	Serine protease	Li et al., 2008
		*Amycolatopsis thailandensis* CMU-PLA07^T^	Natural park soil	Basal medium with PLA film	Plate count and clear zone	–	–	Chomchoei et al., 2011
		*Amycolatopsis oliviviridis* SCM_MK2-4	Agricultural soils	Emulsified PLA agar	Plate count and clear zone	Turbidity method	Protease, esterase and lipase	Penkhrue et al., 2015, 2018
	*Saccharothrix*	*Saccharothrix (Lentzea) waywayandensis*	^∗^JCM 9114	–	–	Film-weight loss; monomer production	Protease	Jarerat and Tokiwa, 2003; Nair et al., 2012
	*Kibdelosporangium*	*Kibdelosporangium aridum*	^∗^JCM 7912	–	–	Film-weight loss; monomer production	Protease	Jarerat et al., 2003
	*Pseudonocardia*	*Pseudonocardia alni* AS4.1531^T^	Chinese culture collection	Basal medium with 0.1% (w/v) gelatin and 50 mg PLA films	–	Film-weight loss; monomer production	–	Konkit et al., 2012
		*Pseudonocardia* sp. RM423	Kasetsart University, Thailand	Emulsified-PLA	Clear zone	Film-weight loss; CO_2_ content	–	Apinya et al., 2015
	*Streptoalloteichus*	*Streptoalloteichus* sp.	^∗^JCM and ^∗∗^IFO	Emulsified-PLA and silk fibroin agar	Plate count and clear zone	Total organic carbon (TOC); residual films in the culture broth	–	Jarerat et al., 2002
*Streptomycetaceae*	*Streptomyces*	*Streptomyces* sp. APL3	Compost soils	Emulsified PLA agar	Plate count and clear zone	–	Serine hydrolase	Sriyapai et al., 2018
		*Streptomyces* sp. KKU215	Botanical garden soil	Emulsified PLA agar	Plate count and clear zone	PLA-packaging weight loss and surface change	–	Yottakot and Leelavatcharamas, 2019
*Streptosporangiaceae*	*Thermopolyspora*	*Thermopolyspora*	Compost	–	Molecular technique	–	–	Sangwan and Wu, 2008
		*Thermopolyspora flexuosa* FTPLA	Compost	Mineral agar media with PLA suspension	Plate count and TGGE method	–	–	Husárová et al., 2014
	*Nonomuraea*	*Nonomuraea terrinata* L44-1	Soil	Emulsified PLA agar	Plate count and clear zone	Turbidity method	–	Sukkhum et al., 2009b
		*Nonomuraea fastidiosa* T9-1	Soil	Emulsified PLA agar	Plate count and clear zone	Turbidity method	–	Sukkhum et al., 2009b
*Thermomonosporaceae*	*Thermomonospora*	*Thermomonospora*	Compost	–	Molecular technique	–	–	Sangwan and Wu, 2008
		*Thermobifida alba* AHK119	Compost	–	–	–	Cutinase	Hu et al., 2010; Kitadokoro et al., 2019
	*Actinomadura*	*Actinomadura keratinilytica* T16-1	Soil	Emulsified PLA agar	Plate count and clear zone	Turbidity method	Serine protease	Sukkhum et al., 2009b
		*Actinomadura* sp. TF1	Compost soils	Emulsified PLA agar	Plate count and clear zone	–	Serine hydrolase	Sriyapai et al., 2018

*^∗^Obtained from JCM (Japan Collection of Microorganisms); ^∗∗^Obtained from IFO (Institute for Fermentation, Osaka).*

The most dominant PLA degrading actinobacteria are members of the genus *Amycolatopsis* namely *Amycolatopsis* sp. HT-32 (Pranamuda et al., 1997), *Amycolatopsis* sp. 3118 (Ikura and Kudo, 1999), *Amycolatopsis* sp. KT-s-9 (Tokiwa et al., 1999), *Amycolatopsis mediterranei* ATCC 27649 (Pranamuda and Tokiwa, 1999), *Amycolatopsis* sp. 41 (Pranamuda et al., 2001), *Amycolatopsis* sp. K104-1 (Nakamura et al., 2001), *Amycolatopsis orientalis* ssp. *orientalis* (Li et al., 2008), *Amycolatopsis thailandensis* CMU-PLA07^T^ (Chomchoei et al., 2011), and *Amycolatopsis* sp. SCM_MK2-4 (Penkhrue et al., 2015). Members of the genus *Amycolatopsis* are well known producers of secondary metabolites which can be exploited for biotechnological applications (Tan and Goodfellow, 2015; Sangal et al., 2018). *Amycolatopsis* species are commonly found in arid or hyper-arid soils (Tan and Goodfellow, 2015) as exemplified by the description of recent novel *Amycolatopsis* from desert soils (Busarakam et al., 2016; Idris et al., 2018). However, these sources have been explored only for bioactive compounds producing *Amycolatopsis.* We opine that arid and hyper-arid environments might harbor diverse *Amycolatopsis* strains with potential PLA degrading properties.

Recently, research on PLA-degrading actinobacteria is focus on the mechanisms of degradation and their enzymatic role. The success of such studies relies on the ability to obtain these PLA-degraders in pure culture under laboratory conditions. In general, there are three approaches for the selective isolation of PLA degrading actinobacteria from environmental samples. The first approach is a simple direct isolation from the samples. Environmental samples are taken, prepared for an appropriate serial dilution and spread directly on emulsified PLA agar. After incubation at suitable temperature, plates were observed for the clear zone around colonies as an indicator for PLA degradation. For instance, the first PLA degrading actinobacterium in the family *Pseudonocardiaceae*, *Amycolatopsis* sp. HT-32 was isolated from soil samples and reported to form clear zone around colonies on emulsified PLA plate within 14 days (Pranamuda et al., 1997). Many PLA degrading actinobacteria were isolated from various environmental samples by dilution spread plate on emulsified PLA agar using this approach including a novel species, *Amycolatopsis oliviviridis* (Table 1). This direct isolation plate assay using emulsified PLA provides a convenient and easy way for screening large numbers of isolates for PLA degraders.

The second approach involves repeated adaptation and selection procedures. In this approach, basal agar plates were seeded with samples then, overlaid with PLA film. Colonies growing and appearing on the PLA films were selected and cultured in 10 mL basal medium containing 0.2% (w/v) PLA film at 30°C for 8 weeks to observe PLA degradation (Ikura and Kudo, 1999). The strains that decreased the film weight by more than 5.5% were transferred to a new liquid medium and incubated at 30°C and 37°C for 4 weeks, and strains that efficiently decreased the film weight were isolated. This method allows adaptation of actinobacteria to PLA degradation as PLA is a synthetic polymer generally not available in nature. Highly efficient PLA-degrading strains could be obtained by this adaptation approach as exemplified in the recent report on the degradation of PLA packaging by *Streptomyces* sp. KKU215 (Yottakot and Leelavatcharamas, 2019).

The third approach involved an enrichment step. Enrichment method is a very useful microbiological tool which is used to support the growth of interested microbes and to increase their small number in environmental samples to detectable levels (Pham and Kim, 2012). Briefly, a soil sample is cultured in a basal medium containing 0.1% (w/v) PLA film before the sample-enriched culture broth was spread onto the emulsified PLA agar. Novel actinobacteria, *Amycolatopsis thailandensis* CMU-PLA07^T^ has been successfully isolated from natural park soil sample in northern Thailand using this approach (Chomchoei et al., 2011).

Over the past two decades, researchers have relied on conventional isolation methods to search for PLA degrading microorganisms in particular actinobacteria. However, it is well accepted that only a small fraction (approximately 1%) of bacteria exists in nature can be grown in the laboratory (Pham and Kim, 2012; Stewart, 2012). Modern molecular biological techniques provide a powerful tool to explore diversity of PLA degrading microorganisms in the environments. Most available techniques involve the analysis of nucleic acids extracted directly from the environments to study microbial diversity, thus avoid limitations of the culture dependent approach. This culture independent approach offers a useful tool for the detection of new group of PLA degrading actinobacteria.

Additional genera of PLA-degrading actinobacteria were found from compost samples using culture independent approach (Sangwan and Wu, 2008; Sangwan et al., 2009; Husárová et al., 2014). Total genomic DNA were extracted from the prepared mixtures sample and amplified by polymerase chain reaction (PCR) of small-subunit rRNA gene sequences to compare microbial community in compost before (day 0) and after PLA degradation (days-60) (Sangwan and Wu, 2008). Members of uncultured bacterial phyla at day 0 are mixture of Actinobacteria, Bacteroidetes, Chloroflexi, Firmicutes, Gemmatimonadetes, Planctomycetes, Proteobacteria, and TM7 while Actinobacteria are dominant in day-60 sample. The majority of cloned gene sequences identified belonged to members of the phyla Actinobacteria in the genera *Actinomadura*, *Amycolatopsis, Pseudonocardia, Salinispora, Thermomonospora*, and *Thermopolyspora.* Actinobacteria especially those belonging to the genera *Thermopolyspora* and *Thermomonospora*, are suggested to have important roles in biodegradation of PLA under composting conditions as these two genera were subsequently isolated from the compost sample at the end of the experiment. The PLA degradation under aerobic composting was observed as a change in PLA molecular weight and polydispersity index (PDI, ${\bar{M}}_{\text{W}}/{\bar{M}}_{\text{n}}$). After 60 days of composting, the ${\bar{M}}_{\text{n}}$ of PLA was reduced from 1.3 × 10^5^ to approximately 5564 Da. The PDI comparison of PLA sample before and after 60 days of composting was slightly decreased (before composting, ${\bar{M}}_{\text{W}}/{\bar{M}}_{\text{n}}$=1.5; after 60 days composting, ${\bar{M}}_{\text{W}}/{\bar{M}}_{\text{n}}$=1.1) (Sangwan and Wu, 2008).

Cultivation experiment on the same day-60 compost found that most bacterial isolates selected for identification were belonged to the genera *Rhizobium*, *Bacillus*, and *Tuberibacillus*, with only two actinobacterial isolates belonged to the genera *Thermomonospora* and *Thermopolyspora*. The discrepancy between cultivation and molecular studies strongly suggested that culture dependent and independent approaches should be used in combination to search for new PLA degrading microorganisms from environmental samples. Similar result was reported by Husárová et al. (2014), PLA degrading bacteria identified as *Thermopolyspora flexuosa* FTPLA was isolated from compost and also detected by temperature gradient gel electrophoresis (TGGE) analysis. From these studies, it is evident that compost is also an interesting source for PLA degrading actinobacteria. These works highlight the importance of using novel molecular ecological techniques for *in situ* identification of novel microorganisms involved in biodegradation of polymeric materials in particular PLA and guided the isolation of such organisms in pure culture.

## Actinobacteria as Potential PLA Degrader – Enzymatic Degradation and Inducers

Polymeric materials like a poly(lactic acid) can be degraded by several microorganisms using their secreted enzymes by hydrolysis reaction (Banerjee et al., 2014). Many studies have been conducted on purification of depolymerase enzymes from PLA degrading microorganisms. Key enzymes which play an important role in depolymerization of PLA are carboxylesterase, cutinases, lipases, and serine proteases (Hajighasemi et al., 2016). Interestingly, only serine protease is identified as an important enzyme in PLA-degrading activities of actinobacteria in the genus *Amycolatopsis* (Tokiwa and Jarerat, 2004; Lim et al., 2005; Tokiwa and Calabia, 2006; Kawai, 2010). Serine protease usually follows a two-step reaction of hydrolysis. In the first step, PLA substrate binds to the surface of serine protease at the active site. Second step involves the cleavage of peptide-like bonds in PLA through reaction of catalytic amino acids (Ser, Asp, and His) collaborates with water (Hedstrom, 2002). However, little information on the purification and characterization of PLA degrading enzymes are available.

Purified PLA degrading enzymes have been reported from three PLA degrading *Amycolatopsis*. The first report dealing with the purification of a poly(L-lactic acid) degrading enzyme was purified from mesophilic actinobacteria, *Amycolatopsis* sp. strain 41 with a molecular weight of 40 kDa (Pranamuda et al., 2001). This enzyme is able to degrade PLLA powder, casein, silk fibroin, succinyl-(L-alanyl-L-alanyl-L-alanine)-*p*-nitroanilide (Suc-(Ala)_3_-*p*NA), but not polycaprolactone (PCL) nor polyhydroxybutyrate (PHB) with optimum pH and temperature of 6.0 and 37–45°C, respectively. The enzyme production could be induced by silk powder from silkworm cocoons. These authors suggested that this purified enzyme is a protease with higher PLLA degrading activity than proteinase K.

Extracellular PLA depolymerase from *Amycolatopsis* sp. strain K104-1 was purified and characterized (Nakamura et al., 2001). The purified enzyme can degrade high-molecular-weight PLA in emulsion and solid film to lactic acid. This enzyme also degraded casein and fibrin but did not hydrolyze collagen type I, triolein, tributyrin, PHB, or PCL. It has a molecular weight of 24 kDa with an optimum pH and temperature of 9.5 and 55–60°C, respectively. The PLA degrading activity was inhibited by di-isopropyl fluorophosphates (DFP) and phenylmethanesulphonylfluoride (PMSF) which indicated that this purified enzyme was a serine type protease.

Three novel PLA-degrading enzymes namely PLAase I (24.0 kDa), II (19.5 kDa), and III (18.0 kDa) were purified from *A. orientalis* ssp. *orientalis* (Li et al., 2008). The optimum pH was between 9.5–10.5 and temperature of 50–60°C. These purified enzymes could degrade high molecular weight PLA film as well as casein. The PLA degrading activity of all three enzymes are higher than proteinase K. They were identified as serine-like protease as their PLA degrading activities were strongly inhibited by serine protease inhibitors such as phenylmethylsulfonylfluoride and aprotinin. In addition, N-terminal amino acid sequence of the protein was determined for the initial 17 residues (IIGGSNATSGPYAARLF). Seven N-terminal amino acid residues were 100% identical to fibrinolytic proteases from the earthworm while ten N-terminal amino acid residues also revealed 80% identical to collagenolytic serine proteases from the hepatopancreas of the Kamchatka crab (Nakamura et al., 2001). From these results, purified depolymerase enzymes from PLA degrading *Amycolatopsis* strains are classified as serine protease which is specific to poly(L-lactic acid) (Matsuda et al., 2005; Tokiwa and Calabia, 2006; Kawai, 2010; Kawai et al., 2011; Wierckx et al., 2018).

Polylactic acid-degrading enzyme was also purified from *Actinomadura keratinilytica* T16-1 with molecular weight of 30 kDa is able to degrade Suc-(Ala)_3_-*p*NA, gelatin, PLA and casein but not PCL (Sukkhum et al., 2009b). The optimum pH and temperature were 10.0 and 70°C, respectively. N-terminal amino acid sequence of the purified enzyme was determined for the initial 15 residues (GYQNNPPSAGLDRAA). The hydrolyzing activity of the purified enzyme was inhibited by DFP, EDTA, and PMSF. This observation indicated that PLA-degrading enzyme produced from *A. keratinilytica* T16-1 is serine protease.

It has been reported that several actinobacteria exhibited a high level of PLLA-degrading activity when liquid culture medium were supplemented with poly(amino acids) inducers such as silk fibroin, elastin, gelatin, some peptides, and amino acids (Jarerat et al., 2004; Tokiwa and Calabia, 2006). For example, the PLLA-degrading enzyme activity of *A. orientalis* was increased from 0 U/mL to 450 U/mL in the medium supplemented with silk fibroin (0.1% w/v). Similarly, the supplement of 0.1% (w/v) elastin was able to increase the PLA-degrading enzyme activity of *Lentzea waywayandensis* (formerly *Saccharothrix waywayandensis*) to 96 U/mL compared to culture control without inducer (0 U/mL) (Jarerat et al., 2004). Most inducers contain L-alanine which is similar to L-lactic acid subunits of PLA in the stereochemical position of chiral carbon (Qi et al., 2017). For example, the PLLA-degrading activity of *Amycolatopsis* sp. 41 was increased with the addition of silk powder into the culture medium from 8 mg TOC/h/mL to 258 mg TOC/h/mL within 5 days (Pranamuda et al., 2001). PLLA-degrading enzymes of *L. waywayandensis* and *Kibdelosporangium aridum* were capable of degrading high molecular weight PLA film (${\bar{M}}_{\text{n}}$: 3.4 × 10^5^) in a liquid medium containing 0.1% (w/v) gelatin (Jarerat and Tokiwa, 2003; Jarerat et al., 2003).

The induction of PLA-degrading enzyme activities of two actinobacteria *L. waywayandensis* and *A. orientalis* was investigated using various poly(L-amino acids) (silk fibroin, gelatin, elastin, and keratin), peptides [(Ala)_2_, Ala-Gly, (Gly)_2_, (Gly)_3_, Gly-Ala, and (Gly)_2_-Ala] and amino acids (alanine, glycine, serine leucine, lysine, methionine, and valine) (Jarerat et al., 2004). The enzyme activities were varied between different strains and inducers. Silk fibroin was the best inducer for *A. orientalis* and elastin for *L. waywayandensis*. A clean biological recycling of PLA under mild condition (40°C) using enzyme from *A. orientalis* IFO12362^T^ without organic solvent was also proposed (Jarerat et al., 2006). However, repolymerization of the degradation products was not carried out. *Pseudonocardia alni* AS4.1531^T^ was also reported to respond to 0.1% (w/v) gelatin inducer (Konkit et al., 2012). Similar result was observed in *Pseudonocardia* sp. RM423 in liquid medium with 0.3% (w/v) gelatin as an inducer (Apinya et al., 2015). Strain RM423 was augmented into soil to assist the indigenous microorganisms with PLA degradation under both mesophilic and thermophilic conditions.

## Development and Application of PLA Degradation by Actinobacteria

Development of microbial-degradation process and formulation of PLA-degrading enzyme production media are needed as a sustainable technology for PLA waste management. Single culture, co-culture and microbial consortium were tried out to find the most effective biodegradable conditions. A comprehensive study on development of actinobacteria for PLA-degradation was reported in *A. keratinilytica* T16-1. Strain T16-1 is a novel actinobacteria isolated from Thai forest soil that showed the highest PLA-degrading activity at high temperature (50°C) in liquid medium supplemented with PLA film as a carbon source (Sukkhum et al., 2009b, 2011). The fermentation processes for PLA degrading enzyme production by strain T16-1 has been well-studied. Biodegradation efficiency of strain T16-1 was investigated in shake flask culture. Factors affecting the enzyme production by *A. keratinilytica* T16-1 and medium optimization were investigated using response surface methodology (Sukkhum et al., 2009a). Poly(L-lactide)-degrading enzyme production by this strain was carried out in a 3L airlift fermenter under the obtained optimum conditions. The optimal conditions of crude enzyme production were demonstrated as 0.43 vvm aeration rate, pH 6.85 and temperature of 46°C. The obtained enzyme showed potential for PLA polymer recycle as L-lactic acid was found as the major degrading product. A yield of 800 mg/L L-lactic acid was obtained from the degradation of 4,000 mg/L L-PLA powder at 60°C for 8 h (Sukkhum et al., 2012).

In addition, re-polymerization of PDLLA recycling process was evaluated by statistical methods based on central composite design (CCD) to develop new technologies for reducing plastic waste in the near future. Approximately 6,700 mg/L PLA powder was degraded by the crude enzyme under optimized conditions (Youngpreda et al., 2017). The degradation products were re-polymerized to form a PLA oligomer (${\bar{M}}_{\text{W}}$ 378 Da). PLA-degrading enzyme activity of *A. keratinilytica* T16-1 was improved and reached 150 U/mL within 72 h in an up-scale 3L airlift fermenter (Panyachanakul et al., 2017). Furthermore, a suitable immobilization material and optimum medium for PDLLA-degrading enzyme production by *A. keratinilytica* T16-1 was investigated. A scrub pad gave the best performance, giving a crude enzyme activity of 30.03 U/mL in shake flask culture. PDLLA-degrading enzyme production of 766.33 U/mL with 15.97 U/mL⋅h of enzyme productivity were achieved with the optimum fermentation conditions of 0.25 vvm aeration, 170 rpm agitation and 45°C incubation temperature for 48 h in batch fermentation using 5L stirrer fermenter. Recently, a scale-up for PLA degrading enzyme production by *A. keratinilytica* T16-1 in a 5L stirred tank bioreactor was carried out under batch condition (Panyachanakul et al., 2019). The best condition for PLA degradation was an agitation speed of 50 rpm at 60°C under a controlled pH of 8.0. A potential method for commercial PLA material degradation by this crude enzyme was also reported. However, lactic acid as degradation products showed inhibitory effect on PLA degradation process. PLA degradation efficiency could be enhanced using simultaneous PLA degradation and dialysis method. The maximum conversion efficiency (percentage of PLA that had been degraded to liberated lactic acid) of 89% was achieved after incubation for 72 h under optimum condition.

Polylactic acid recycling methods such as chemical hydrolysis and pyrolysis have been previously reported (Tsuji and Nakahara, 2002; Fan et al., 2003). However, biological recycling process is considered to be a more environmentally friendly and sustainable approach for PLA waste management. PLA degrading enzymes from actinobacteria such as *A. orientalis* (mesophilic condition) and *A. keratinilytica* T16-1 (thermophilic condition) are proved to be a potential candidate for biological recycling of PLA. Large scale fermentation of PLA degrading enzyme as exemplified in *A. keratinilytica* T16-1 is opened up the opportunity for a sustainable PLA waste management.

## Genomic Insights Into PLA-Degrading Actinobacteria of the Genus *Amycolatopsis*

The genome sequences of six representatives *Amycolatopsis* strains, namely *A. alba* DSM 44262 (accession number: KB913032), *A. balhimycina* DSM 44591^T^ (accession number: AJ508239), *A. japonica* MG417-CF17 (accession numbers: CP008953), *A. orientalis* B-37 (accession numbers: CPCC200066), *A. mediterranei* U32 (accession numbers: CP002000), and *A. thailandensis* JCM 16380^T^ (accession numbers: NZ_NMQT00000000.1) were obtained from GenBank. These six phylogenetically closely related strains were selected based on whole genome comparison (data not shown) to test whether comparative genomics could provide useful information for the selection of PLA degrading strains. *A. tolypomycina* DSM 44544^T^ (accession numbers: NZ_FNSO00000000) was also included in an analysis as a distantly related strain with the six *Amycolatopsis* strains. *A. alba*, *A. orientalis*, *A. mediterranei*, and *A. thailandensis* have been previously reported to have PLA degrading ability (Pranamuda and Tokiwa, 1999; Li et al., 2008; Chomchoei et al., 2011). All sequences were annotated using the Rapid Annotation Subsystem Technology (RAST) server (Aziz et al., 2008). The distribution of PLA-degrading genes in the genomes was determined using the SEED server (Overbeek et al., 2014) with focus on genes encoding for protein degradation, serine endopeptidase and those regulating the effect of PLA-degrading activities. Genomic features of these six *Amycolatopsis* strains were listed in Table 2.

**TABLE 2:** General features of the genome of representative *Amycolatopsis* species.

**Species/Strains**	**Accession number**	**Genome size (bp)**	**Contigs**	**No. of coding sequences**	**tRNAs**	**G + C content (%)**
*Amycolatopsis alba* DSM 44262^T^	KB913032	9,811,274	1	9228	62	68.7
*Amycolatopsis balhimycina* DSM 44591^T^	AJ508239	10,858,503	1	10190	65	70.8
*Amycolatopsis japonica* MG417-CF17	CP008953	8,961,318	1	8532	67	68.9
*Amycolatopsis mediterranei* U32	CP002000	10,236,715	1	9946	63	71.3
*Amycolatopsis orientalis* B-37	CPCC200066	9,490,992	1	8647	62	68.8
*Amycolatopsis thailandensis* JCM 16380^T^	NZ_NMQT00000000.1	9,348,263	421	8990	67	68.8
*Amycolatopsis tolypomycina* DSM 44544^T^	NZ_FNSO00000000	10,363,431	4	9780	64	71.7

Serine proteases/serine endopeptidases (EC 3.4.21.-) are enzymes responsible for PLA degradation. It was purified and characterized from PLA-degrading actinobacteria, *Amycolatopsis* sp. (Pranamuda et al., 2001; Li et al., 2008). Comparative genomics reveal that serine endopeptidase related genes such as *sspA* and *blaSE* are present in protein degradation subsystem category of all representatives *Amycolatopsis* species (Supplementary Table 1). However, there is no report on PLA degrading activities of either *A. balhimycina* DSM 44591^T^ or *A. japonica* MG417-CF17. It is reasonable to believe that these two species will have PLA degrading property similar to their phylogenetically related neighbors. Interestingly, these genes encoding for PLA degrading enzymes are also present in genomes of *A. tolypomycina* DSM 44544^T^ which is distantly related to the six representative strains. From this small set of comparison, it seems that these genes related to PLA degradation are conserved within the members of the genus *Amycolatopsis*. We believe that this genomic analysis will provides a powerful tool for exploiting genetics potential of actinobacteria for PLA degradation.

## Conclusion and Future Perspectives

This mini-review provides evidence to support the view that actinobacteria are potential PLA degrading microorganisms. Several members of actinobacteria are able to degrade poly(lactic acid) bioplastics either under laboratory conditions or under field trials (Table 1). Selective isolation and cultivation of PLA degrading actinobacteria under laboratory conditions is proving to be a challenge. There is still a room for improvement in selective isolation procedures and strategies to target specific actinobacterial taxa of interest. A sound taxonomic data will provide a ground for an improved isolation and cultivation for PLA degrading actinobacteria. Comparative genomics offer a tool for screening of potential PLA degrading actinobacteria as exemplified in this study. The accumulation of high-quality whole genome data provides a useful information to support the search for novel actinobacterial strains with PLA degrading ability. Actinobacteria are well known for their ability to produce bioactive compounds. However, their potential as PLA degraders is just becoming apparent. PLA degrading actinobacteria such as *Amycolatopsis* and *Actinomadura* have shown promise which deserves research attention as market demand for PLA plastics are continuously on the rise.

## Author Contributions

NB contributed to the data for diversity of PLA degrading actinobacteria, their enzyme production and inducers, whole genome analysis and Tables 1, 2. WP-A conceived the idea, wrote and revised the manuscript.

## Conflict of Interest

The authors declare that the research was conducted in the absence of any commercial or financial relationships that could be construed as a potential conflict of interest.
