# Supplementary material for: Actinobacteria as Promising Candidate for Polylactic Acid Type Bioplastic Degradation
Source: Front Microbiol. 2019 Dec 19;10:2834. doi: 10.3389/fmicb.2019.02834 (PMC6930877; doi:10.3389/fmicb.2019.02834)
Supplement: Supplementary file 1 [file Data_Sheet_1.PDF]

Supplementary Table 1 Enzymatic role on PLA-degradation of *Amycolatopsis* sp. from SEED database.

Microbial species	Category	Sub-category	Subsystem	EC number	Abbrev.	Functional roles
Amycolatopsis alba DSM 44262^{T}	Protein metabolism	Protein degradation	Serine endopeptidase	EC 3.4.21.-	GluXxx	Glutamyl endopeptidase precursor (EC 3.4.21.19), serine proteinase SspA
					GluXxx2	Glutamyl endopeptidase precursor (EC 3.4.21.19), blasé
					ProXxx	Prolyl endopeptidase (EC 3.4.21.26)
					LysXxx	Lysyl endopeptidase (EC 3.4.21.50)
Amycolatopsis balhimycina DSM 44591^{T}	Protein metabolism	Protein degradation	Serine endopeptidase	EC 3.4.21.-	GluXxx	Glutamyl endopeptidase precursor (EC 3.4.21.19), serine proteinase SspA
					GluXxx2	Glutamyl endopeptidase precursor (EC 3.4.21.19), blasé
					ProXxx	Prolyl endopeptidase (EC 3.4.21.26)
					LysXxx	Lysyl endopeptidase (EC 3.4.21.50)
Amycolatopsis japonica MG417-CF17	Protein metabolism	Protein degradation	Serine endopeptidase	EC 3.4.21.-	GluXxx	Glutamyl endopeptidase precursor (EC 3.4.21.19), serine proteinase SspA
					GluXxx2	Glutamyl endopeptidase precursor (EC 3.4.21.19), blasé
					ProXxx	Prolyl endopeptidase (EC 3.4.21.26)
					LysXxx	Lysyl endopeptidase (EC 3.4.21.50)
Amycolatopsis mediterranei U32	Protein metabolism	Protein degradation	Serine endopeptidase	EC 3.4.21.-	GluXxx	Glutamyl endopeptidase precursor (EC 3.4.21.19), serine proteinase SspA
					GluXxx2	Glutamyl endopeptidase precursor (EC 3.4.21.19), blasé
					ProXxx	Prolyl endopeptidase (EC 3.4.21.26)

					LysXxx	Lysyl endopeptidase (EC 3.4.21.50)
Amycolatopsis orientalis B-37	Protein metabolism	Protein degradation	Serine endopeptidase	EC 3.4.21.-	GluXxx	Glutamyl endopeptidase precursor (EC 3.4.21.19), serine proteinase SspA
					GluXxx2	Glutamyl endopeptidase precursor (EC 3.4.21.19), blaSE
					ProXxx	Prolyl endopeptidase (EC 3.4.21.26)
					LysXxx	Lysyl endopeptidase (EC 3.4.21.50)
Amycolatopsis thailandensis JCM 16380^T	Protein metabolism	Protein degradation	Serine endopeptidase	EC 3.4.21.-	GluXxx	Glutamyl endopeptidase precursor (EC 3.4.21.19), serine proteinase SspA
					GluXxx2	Glutamyl endopeptidase precursor (EC 3.4.21.19), blaSE
					ProXxx	Prolyl endopeptidase (EC 3.4.21.26)
					LysXxx	Lysyl endopeptidase (EC 3.4.21.50)
Amycolatopsis tolypomycina DSM 44544^T	Protein metabolism	Protein degradation	Serine endopeptidase	EC 3.4.21.-	GluXxx	Glutamyl endopeptidase precursor (EC 3.4.21.19), serine proteinase SspA
					GluXxx2	Glutamyl endopeptidase precursor (EC 3.4.21.19), blaSE
					ProXxx	Prolyl endopeptidase (EC 3.4.21.26)
					LysXxx	Lysyl endopeptidase (EC 3.4.21.50)
